# High-transmission acoustic self-focusing and directional cloaking in a graded perforated metal slab

**DOI:** 10.1038/s41598-017-04512-z

**Published:** 2017-06-29

**Authors:** Sheng-Dong Zhao, Yue-Sheng Wang, Chuanzeng Zhang

**Affiliations:** 1 Institute of Engineering Mechanics, Beijing Jiaotong University, Beijing, 100044 China; 2 Department of Civil Engineering, University of Siegen, Siegen, 57068 Germany

## Abstract

A design strategy and its modeling for high-transmission acoustic self-focusing and directional cloaking in a two-dimensional (2D) and an axisymmetric three-dimensional (3D) gradient-index phononic crystal (GRIN-PC) are reported in this paper. A gradient perforated aluminum slab sandwiched by water is considered. A low-loss directional cloaking device is achieved by controlling the matching coefficient between the slab and the water. The anisotropy coefficient that affects the scattering properties is also introduced. Furthermore, the phase discontinuity for directional cloaking inside and outside the slab is overcome by introducing a non-gradient slab having a lower group velocity behind the GRIN slab as an acoustic delay device. In addition, an axisymmetric 3D directional cloaking structure is obtained by rotating the corresponding 2D structure around the slab axis.

## Introduction

Optical or acoustical invisibility devices can keep objects undetectable to electromagnetic or sound waves. Cloaking is a fascinating subject that attracts many researchers’ attention in recent years. This kind of devices attains a rapid development thanks to the transformation optics (TO) methodology^1^ and the metamaterial technology^2–4^, which are initially applied to electromagnetic waves and then extended to other waves^5–10^. Pentamode metamaterials having an unconventional anisotropic stiffness and isotropic mass density are appropriate to the cloaking design^11–13^. Scattering cancellation^14–16^ is a prominent alternative that allows for a broadband and omni-directional performance. A structure on sub-wavelength scales is required for the cloaking devices. Since acoustic wavelengths at a scale of meters are typically larger than optical wavelengths at a scale of microns, the acoustic cloaking can be easily realized.

Perfect cloaking based on the TO method provides a possibility to make the object invisible even for sensitive detectors. But the required large and precisely controlled anisotropy of the refractive index^17^ is difficult to fabricate. Due to the resonance specifics and losses in traditional metamaterials, the TO-based cloaks are of a narrow band and the cloaking size is rather limited^18, 19^. Therefore, many other ways are proposed for invisibility without employing metamaterials^20–24^. Yamada *et al*.^25^ proposed a topology optimization method for a dielectric optical cloak. Directional cloaks, as proposed by Leonhardt^26^, are the devices that can offer cloaking for a specified propagation direction. Urzhumov and Smith^23^ showed how to take the advantage of the phase delay to achieve a limited form of the directional invisibility that does not require metamaterials. Duan *et al*.^24^ used the geometrical optical principles to develop a unidirectional transmission cloak for hiding objects that substantially exceed the incident radiation wavelengths. The versatility of acoustic devices can be achieved by phononic crystals (PCs). And the control of the acoustic wave propagation can be further enhanced by introducing a GRIN-PC. With appropriately designed gradient, the GRIN-PC can be used for focusing^27–30^, wave bending^31, 32^, beam shifting, and acoustic cloaking. Martin *et al*.^33, 34^ reported experimentally a GRIN lens in water based on phononic crystals, and a particular one that is impedance matched with water. Vasic and Gajic^35^ investigated the realization of the self-focusing using two-dimensional (2D) graded photonic crystals. And a directional cloak was obtained by modifying the self-focusing lens. These previous investigations have demonstrated that the GRIN-PC based devices work well up to the Bragg frequencies.

In this paper, a 2D graded perforated metal slab designed for acoustic self-focusing and directional cloaking is reported. Our design strategy relies solely on gradient-index wave-guiding, in contrast to the anisotropic coordinate transformation or the modal cancellation. The cloaking effect in this study is quite different from the traditional cloaking design because neither the metamaterial nor the TO method is used. The metamaterials are usually arranged in repeating patterns at scales that are smaller than the wavelengths of the phenomena they influence. Under the long wavelength assumption, the acoustic wavelength manipulated by the metamaterials should be at least more than eight times larger than the lattice constant in liquid^36, 37^. In this paper, the longitudinal wave mode is emphasized for an acoustic cloaking device. The concept is closely related to the so-called pentamode material, but it works in the frequency regime of wave scattering and the homogenization limit is broken. Acoustic cloaking based on the TO method has to resort to metafluids with isotropic stiffness but anisotropic density, which is very hard to manufacture. The present directional cloaking is designed based on the GRIN-PC that can control the incident wave to propagate around the cloaking area. The cloaking performance is properly described by an analytical model based on the numerical simulations. The GRIN-PC based cloaking slab is still narrow banded, but the cloaking size reaches three times of the wavelength in the wave propagation direction and can be amplified by increasing the slab size. Interestingly, by rotating the 2D system around the slab axis, a 3D directional cloaking device is obtained.

## Results

### Problem statement

**Figure 1:**
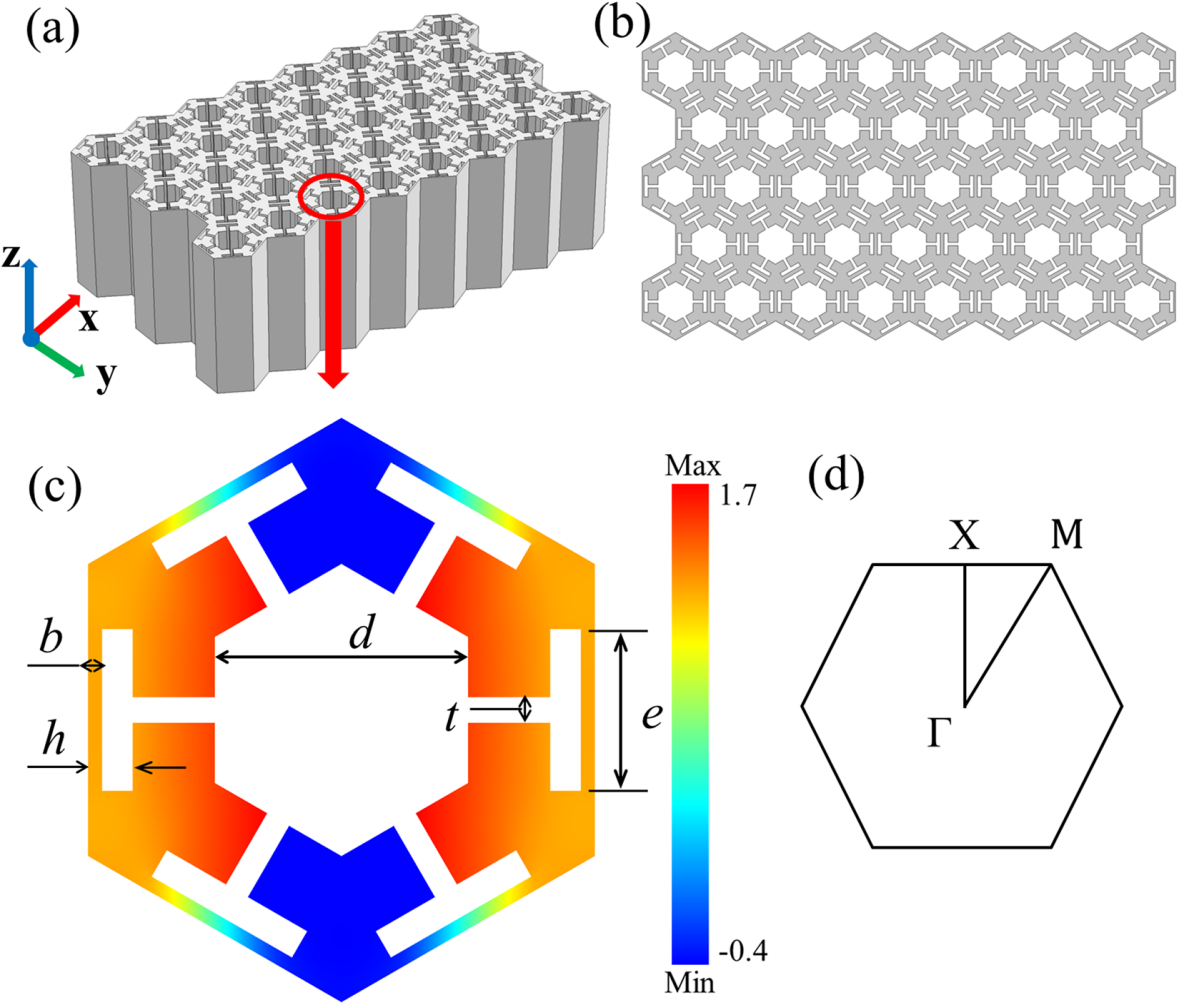
(**a**) The 3D view of the periodic structure; (**b**) the 2D topological structure of the slab; (**c**) the unit-cell of the 2D PC with the color showing the vibration mode of the first longitudinal band along the ΓX direction; and (**d**) the corresponding irreducible Brillouin zone.

Let us consider an aluminum slab as shown in Fig. 1. The slab is sandwiched by water and perforated with a triangular periodic array of complex holes. The special structure of the considered slab results in quasi-isotropic properties in *x*- and *y*-directions.

Figure 1(a) shows the 3D view of the periodic structure, while the 2D top view is presented in Fig. 1(b). The length of the aluminum slab along the *z*-axis is considered to be infinite or sufficiently large, so that the wave propagation in the *x*-*y*-plane can be considered as a 2D problem. The unit-cell of the 2D periodic structure and the corresponding irreducible Brillouin zone are presented in Figs. 1(c) and (d). Without loss of generality, the lattice constant is taken as *a* = 20 mm and the geometry of the unit-cell is characterized by five parameters *b*, *d*, *e*, *h* and *t*, where four of them are fixed as *b* = 0.0275*a*, *e* = 0.32*a*, *h* = 0.0875*a* and *t* = 0.05*a*. In contrast, the parameter *d* is variable so that we can design a GRIN-PC slab with a desired target acoustic index. Hence, the porosity of the perforated slab can be calculated as

1 $$f={(\frac{d}{a})}^{2}-\frac{\sqrt{3}d}{10a}+{\rm{0.2759.}}$$

The used material parameters are as follows: the mass density *ρ* = 2.7 g/cm^3^, the longitudinal sound velocity *c*_*l*_ = 6.15 km/s, and the transverse sound velocity *c*_*t*_ = 3.1 km/s for the aluminum; and the density *ρ*_*0*_ = 1 g/cm^3^, the wave velocity *c*_0_ = 1.49 km/s for the water. The unit-cell composed of the basic honeycomb frame and blocks on the corners can easily generate longitudinal wave modes, and the acoustic properties can match with those of the water^38^. The filling ratio of the geometrical configuration as presented in Fig. 1 can be adjusted to a high degree of freedom without changing the basic honeycomb frame. The block on the corner is divided into two branches and the dispersed distribution can broaden the adjustable range of the filling ratio.

Based on the periodically perforated slab as shown in Fig. 1, self-focusing and directional cloaking devices are constructed, which are described and discussed in the following sections.

### The self-focusing design

A refractive index profile in the form of a hyperbolic secant is chosen for acoustic self-focusing^28, 31^. The refractive index along the transverse direction (*y-*axis) for the GRIN medium is defined as

2 $${n}_{y}={n}_{0}\,{\rm{sech }}(\delta y),$$

where *δ* is the gradient coefficient and *n*_0_ is the refractive index of the central layer (*x-*axis). All refractive indexes in this paper are defined relative to the water. When ignoring the anisotropy of the PC with the designed parameter *d*, the refractive index *n*_*y*_ is written as

3 $${n}_{y}=\bar{k}/{k}_{B},$$

where $\bar{k}=({k}_{{\rm{\Gamma }}{\rm{X}}}+{k}_{{\rm{\Gamma }}{\rm{M}}})/2$ is the average magnitude of the wave vectors of the PC and *k*_*B*_ is that of the background water. The self-focal length for the hyperbolic secant refractive index medium depends only on *δ* through

4 $$L=\pi /(2\delta ).$$

In this model, the focal length is set as *L* = 11*a*, and the initial refractive index *n*_0_ is assumed to be 1.161 corresponding to the parameter *d* = 0.5*a* at the frequency of 25 kHz. Then based on Equations (2) and (3), the wave vector magnitude along the *y-*axis is obtained and the variable parameter *d* is calculated numerically.

**Figure 2:**
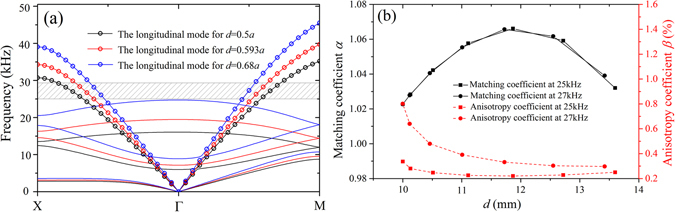
(**a**) The band structures for three different values *d* = 0.5*a*, 0.593*a* and 0.68*a*; and (**b**) the variations of the coefficients *α* and *β* with *d* at the self-focusing frequencies of 25 kHz and 27 kHz.

The band structures for three different values *d* = 0.5*a* (*y* = 0), $0.593a\,(y=2\sqrt{3}a)$ and $0.68a\,(y=3\sqrt{3}a)$ are plotted in Fig. 2(a), where the longitudinal mode is marked by circles. The longitudinal vibration mode along the ΓX direction in the first longitudinal band at the frequency of 25 kHz for the parameter *d* = 0.5*a* is plotted in Fig. 1(c).

By properly selecting the values of the variable parameter *d* in 0.5*a~*0.7*a*, we can get a lens showing a self-focusing frequency between 25 kHz and 28 kHz, see the shadowed region in Fig. 2(a). For any self-focusing frequency, we can calculate $\bar{k}$ and *n*_*y*_ for the unit-cell in an arbitrary *y*-direction based on the dispersion relations *ω*(*k*). The variable geometric parameter of the unit-cell in the *y*-direction is denoted by *d*_*y*_. Firstly, the value of this geometric parameter is evenly selected between 0.5*a* and 0.7*a* and the *i*th value is defined by *d*_*i*_. Then the corresponding refractive index *n*_*i*_ is calculated based on the dispersion relations. Affirmatively, we can find a number “*m*” so that ${n}_{m} < {n}_{y} < {n}_{m+1}$ is satisfied. Based on the linear interpolation, the parameter *d*_*y*_ is obtained as

$${d}_{y}=\frac{{n}_{y}}{{n}_{m}}{d}_{m}.$$

High acoustic transmission is necessary for self-focusing and directional cloaking, which can be characterized by the acoustic matching coefficient between the water and the slab defined by ref. 32

5 $$\alpha =\frac{{\rho }_{e}{c}_{g}}{{\rho }_{0}{c}_{0}},$$

where ${\rho }_{e}=(1\,-\,f)\rho$ is the average density of the perforated PC slab, and ${c}_{g}=({\rm{d}}\omega /{\rm{d}}{k}_{{\rm{\Gamma }}{\rm{X}}}+{\rm{d}}\omega /{\rm{d}}{k}_{{\rm{\Gamma }}{\rm{M}}})/2$ is the average group velocity at the considered frequency in the first longitudinal band. The change of the geometric parameter *d* can induce an opposite variation trend between the average density and the group velocity, which is conductive to keep the matching coefficient at a relatively stable value. Since the parameter *t* significantly affects the porosity and the average density, especially when the parameter *d* decreases, its adjustment provides the most effective way to control the matching coefficient. Through a proper choice of *t*, it can be ensured that the initial value of the matching coefficient is around 1, which implies a high acoustic transmission. So in order to get a pure longitudinal mode and a high acoustic transmittance, the geometrical design in Fig. 1 is very reasonable. The anisotropy coefficient of the PC is defined by

6 $$\beta =\frac{{k}_{{\rm{\Gamma }}{\rm{X}}}\,-\,{k}_{{\rm{\Gamma }}{\rm{M}}}}{{k}_{{\rm{\Gamma }}{\rm{M}}}}.$$

**Figure 3:**
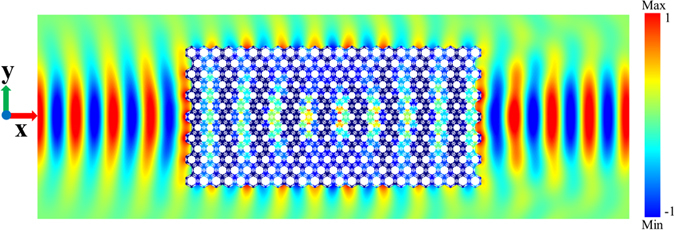
The self-focusing of the GRIN slab at the frequency of 25 kHz.

Based on the adjustment of the parameter *d* of the GRIN-PC, the acoustic self-focusing at different frequencies can be realized. In order to satisfy Equation (2), the anisotropy coefficient *β* should be as small as possible. And by controlling the matching coefficient $\alpha \approx 1$, the low reflection target is achieved. Figure 2(b) shows the variations of the two coefficients *α* and *β* with the design parameter *d* of the two GRIN slabs corresponding to the self-focusing frequencies of 25 kHz and 27 kHz. The horizontal coordinate shows the distribution of the parameter *d* along the *y-*axis. Both of the matching coefficients are slightly larger than 1. However, the anisotropy coefficient of the GRIN slab at the self-focusing frequency 25 kHz is smaller, yielding a better self-focusing, see Fig. 3. The self-focal length from our simulation is 12*a*, which is slightly larger than the theoretical value of 11a. This discrepancy is introduced by the anisotropy (*β* ≠ 0) and the gradient discontinuity. The commercial software COMSOL Multiphysics is used for the numerical simulation.

### The directional cloaking design

**Figure 4:**
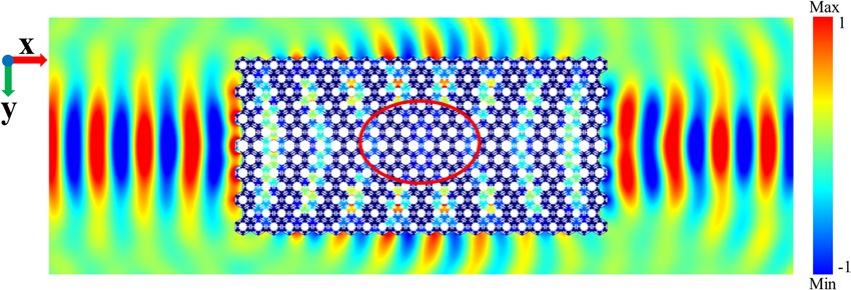
The acoustic wave propagation trajectory in the cloaking slab.

The self-focusing GRIN-PC is then employed for the directional cloaking by exchanging the positions of the center and edge parts of the slab. Therefore, as an inverse conversion process, the incident wave beam with the frequency of 25 kHz will be excluded from the central part of the slab, while once again converged into a beam at the exit (Fig. 4). The cloaking effect relies on a diverging gradient-index profile that is then coupled with the total internal reflection at the upper boundary of the slab. A slightly disturbed area is induced in the central part of the slab (the elliptical area in Fig. 4), which can be used for the object cloaking with an arbitrary shape. Here, a cloaking size of three times of the wavelength is attained in the wave propagation direction, and it can be amplified by increasing the slab size.

**Figure 5:**
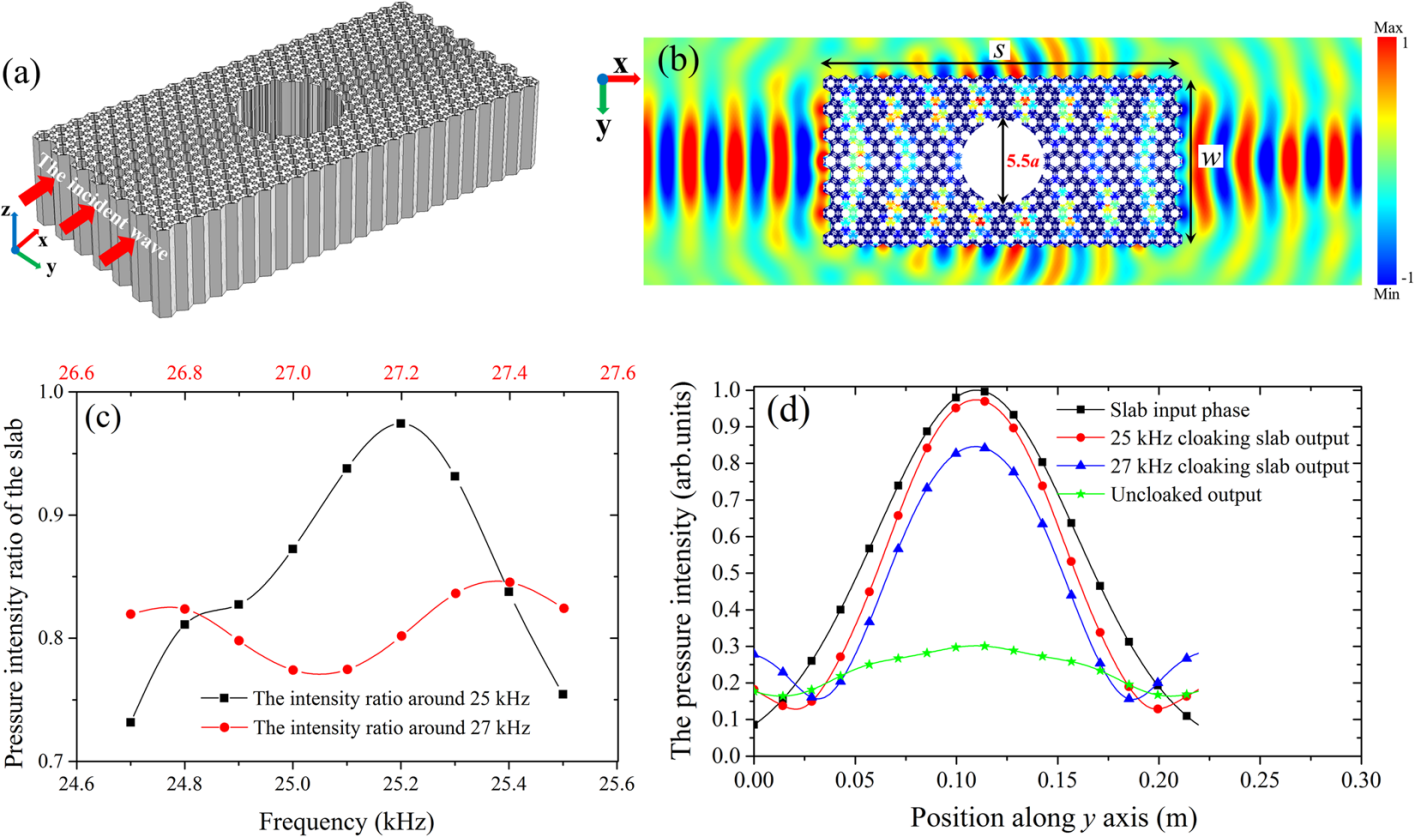
(**a**) The 3D view of the cloaking slab. The circular hole located in the central part is the cloaking area; (**b**) the simulated cloaking result for a circular hole with the diameter of 5.5*a* at the frequency of 25 kHz; (**c**) the ratio of the pressure intensity integrals of the two slabs around their cloaking frequencies of 25 kHz and 27 kHz, respectively; and (**d**) comparison between the normalized pressure intensity vs *y* for the input source position, the 25 kHz cloaking slab output, the 27 kHz cloaking slab output and the uncloaked output wave fields.

The 3D schematic view and the 2D simulation result for the cloaking of a circular hole of the diameter 5.5*a* at the frequency of 25 kHz are shown in Figs. 5(a) and (b). In the present case, the slab width and length are taken as $w=6\sqrt{3}a$ and *s* = 24*a*. The diameter of the cloaking hole accounts for a large region about 53% of the slab width, and the hole has only a small influence on the wave propagation through the slab, see Fig. 5(b). Here, we design two cloaking slabs corresponding to 25 kHz and 27 kHz, respectively. Due to the low mismatch (*a* ≈ 1), the pressure field distributions at the input and output lens faces are similar. Figure 5(c) shows the ratio of the pressure intensity integrals on the output and input faces of the two slabs around their cloaking frequencies of 25 kHz (the black squares) and 27 kHz (the red circles), respectively. The cloaking design of 25 kHz reaches its peak of 0.97 at the frequency of 25.2 kHz, see the black line in Fig. 5(c). Nevertheless, the red line for the cloaking design of 27 kHz is still maintained around 0.8. The higher transmission property is attained for the 25 kHz cloaking slab because it shows a lower anisotropy coefficient which is clarified in Fig. 2(b). To catch the cloaking effect, the similarity of the wave front pattern between the entry and the exit is studied. A comparison between the normalized pressure intensity vs *y* for the input source position and the cloaked and uncloaked output wave field is shown in Fig. 5(d). It is found that the wave front pattern of the 25 kHz cloaking slab is more similar to the entry wave field than that of the 27 kHz cloaking slab. However, both of them are far more efficient than the uncloaked result. In this example, all the cloaking slabs have the same initial refractive index of *n*_0_ = 1.161 and the same gradient coefficient of *δ* = 0.00654/mm.

Obviously, the cloaking size is strongly affected by the slab size. Although the slab length can be increased unlimitedly, the slab width is restricted by the adjustable range of the refractive index of the unit-cell. By substituting Equation (4) into Equation (2), the left boundary position can be written as

7 $$y={\rm{a}}{\rm{s}}{\rm{e}}{\rm{c}}{\rm{h}}(\frac{{n}_{y}}{{n}_{0}})\frac{2L}{\pi }.$$

For the cloaking frequency of 25 kHz, the designed refractive index range based on the variable parameter *d* is $0.868\le {n}_{y}\le 1.161$. If the self-focal length is designed as *L* = 11*a*, the upper limit of the slab width is $w=6\sqrt{3}a$. However, with the self-focal length increasing, the *y*-coordinate reaches a higher value. So the theoretical value of the slab width can be enlarged with the slab length increasing and the same is true for the cloaking size.

**Figure 6:**
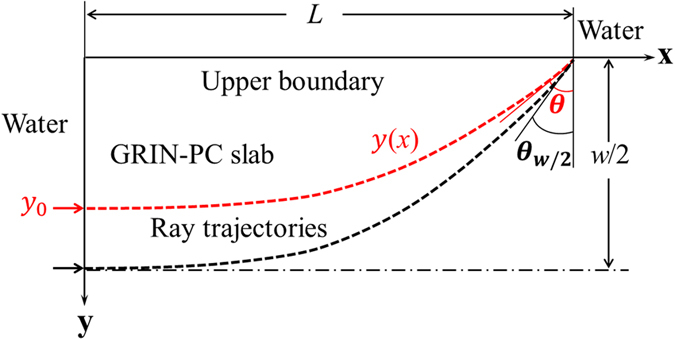
The schematic diagram of the ray trajectory in the cloaking slab.

For preventing the wave to escape out of the boundary of the slab which is determined by the slab length and width, the diverging paths in the slab should be limited within the critical angle of the total reflection from the GRIN-PC to water. This requires that the incidence angle on the upper boundary of the slab should be higher than the critical angle. This can be estimated via a ray trajectory analysis by considering a ray trajectory as shown in Fig. 6, which is incident from the left boundary of the slab and striking the upper boundary at an angle of *θ*. The ray trajectory and its derivative in a GRIN medium with the hyperbolic secant refractive index profile can be analytically derived as^39^

8 $$y(x)=\frac{1}{\delta }{\sinh }^{-1}[{u}_{0}{H}_{f}(x)+{u}_{0}^{^{\prime} }{H}_{a}(x)],$$

9 $${y}^{^{\prime} }(x)=\frac{{u}_{0}{H}_{f}^{^{\prime} }(x)+{u}_{0}^{^{\prime} }{H}_{a}^{^{\prime} }(x)}{\delta \,\cosh \{{\sinh }^{-1}[{u}_{0}{H}_{f}(x)+{u}_{0}^{^{\prime} }{H}_{a}(x)]\}},$$

where $u(x)=sinh[\delta y(x)]$, ${u}^{^{\prime} }(x)=\delta {y}^{^{\prime} }(x)cosh[\delta y(x)]$, ${u}_{0}=u(0)$, ${u}_{0}^{^{\prime} }={u}^{^{\prime} }(0)$; and ${H}_{a}(x)$, ${H}_{a}^{^{\prime} }(x)$, ${H}_{f}(x)$ and ${H}_{f}^{^{\prime} }(x)$ are the position and slop of the axial and field rays given by

10 $${H}_{a}(x)=\frac{\sin (\delta x)}{\delta },{H}_{a}^{{\rm{^{\prime} }}}(x)=\,\cos (\delta x),$$

11 $${H}_{f}(x)=\,\cos (\delta x),{H}_{f}^{^{\prime} }(x)=-\delta \,\sin (\delta x),$$

By substituting $x=L=\pi /(2\delta )$ into Equation (9), the slop of the trajectory intersecting the upper boundary is obtained as ${y}^{^{\prime} }(L)=-sinh(\delta {y}_{0})$ where ${y}_{0}=y(0)$. Then the angle *θ* can be expressed as $\theta =\pi /2-arctan| {y}^{^{\prime} }(L)|$, i.e.,

12 $$\theta =\pi /2\,-\,arctan[sinh(\delta {y}_{0})]=arcsin[{\rm{sech }}(\delta {y}_{0})],$$

where $0\le {y}_{0}\le w/2$ is the incidence location of the ray at the left boundary. So the varying region of the angle *θ* is $\theta \in [{\theta }_{w/2},{90}^{\circ }]$ with ${\theta }_{w/2}=arcsin[{\rm{sech }}(\delta w/2)]$. The critical angle for the total reflection at the upper boundary can be derived by using Snell’s law as

13 $${\theta }_{c}=arcsin(1/{n}_{0}),$$

where *n*_0_ is the refractive index of the cloaking slab on the upper boundary relative to the water. The total reflection condition for all diverging rays can be satisfied when ${\theta }_{w/2} > {\theta }_{c}$, otherwise a certain degree of energy leakage may exist at the boundary. From the condition ${\theta }_{w/2} > {\theta }_{c}$, the total reflection condition in term of the slab size can be determined as

14 $$\frac{w}{L} < \frac{4}{\pi }{\rm{arcsech}}(\frac{1}{{n}_{0}}).$$

It should be pointed out here that the present high-transmission cloaking slab is designed based on multiple mechanisms, namely, the total reflection (${\theta }_{w/2} > {\theta }_{c}$), the impedance matching (*α* = 1), and the anisotropy effect (*β* = 0). If all conditions cannot be strictly satisfied simultaneously in practical applications, then certain slight deviations can be accepted.

Another important issue is the phase discontinuity between the waves traveling inside and outside the slab. The phase matching for the two components of the wave front can be realized by adjusting the difference of the phases inside the cloak and the water space so that ${\rm{\Delta }}\lambda =N{\lambda }_{0}$, where *N* is a positive integer (*N* = 1, 2, 3...) and *λ*_0_ is the water wavelength at the frequency 25 kHz. Unfortunately, the precisely designed GRIN slab cannot meet the above condition exactly. To fulfill this condition, we will introduce an acoustic delay device following the GRIN slab. The group velocity in a periodically perforated slab can be controlled by the properly designed parameter *d*. And this slab without gradient having a particular group velocity is suitable to serve as a delay device. We assume that the phase difference at the exit port of the GRIN slab is ${\lambda }_{e} < {\lambda }_{0}/2$ and the length of the delay device is *l*_0_. Then the group velocity of the delay device satisfies the following condition:

15 $${c}_{g0}=\frac{{l}_{0}{c}_{0}}{{l}_{0}+{\lambda }_{e}}.$$

The length of the delay device is *l*_0_ = 5*a* (corresponding to five layers of the unit-cell). The dispersion relations of the unit-cell with the geometrical parameter *d* can be written as *ω*_*d*_ = *f*(*k*). Based on the band structures, the group velocity at the frequency of 25 kHz is calculated by

16 $${c}_{g0}=\frac{\partial {\omega }_{d}}{\partial k}.$$

**Figure 7:**
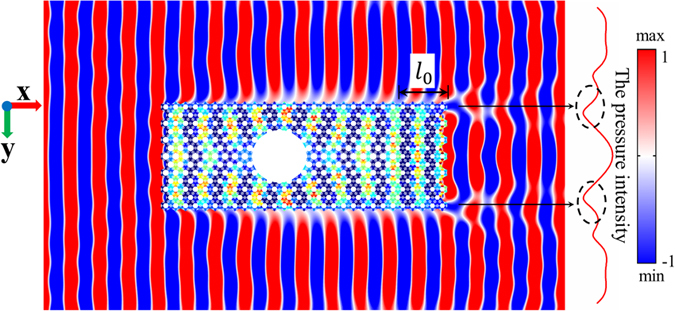
The simulated wave field of the cloaking device with a phase matching by introducing a phase delay device of length *l*_*0*_ at 25 kHz.

By substituting Equation (15) into Equation (16), the geometrical parameter *d* of the delay device can be numerically calculated as 0.475*a* with the matching coefficient *α* slightly larger than 1 which will not affect the high transmission of the slab. The simulated wave field of the cloaking device with a phase matching at the frequency of 25 kHz is shown in Fig. 7. But the wave intensity is not evenly distributed and shows a lower value at the exit port between the wave beams propagating in and out of the slab, see the red line in Fig. 7.

**Figure 8:**
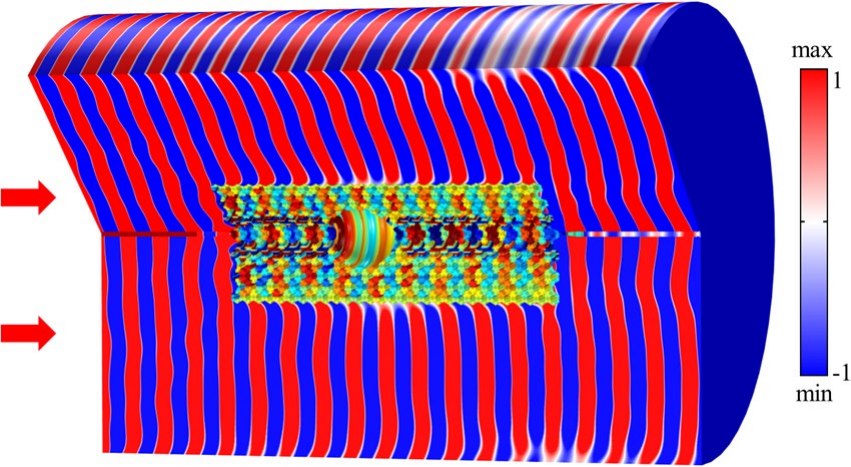
The acoustic pressure field with a spherical cloaking effect in the 3D axisymmetric structure.

The above acoustic cloaking structure is designed using a 2D perforated metal slab. If this 2D system is rotated around the slab axis, a 3D cloaking device formed by the axisymmetric structure can be obtained. A much faster 2D calculation method instead of a 3D one is adopted in this analysis by taking the advantage of the rotational extension method, which has been applied previously to design PC axisymmetric GRIN lenses for realizing acoustic focusing in ref. 40. The acoustic wave propagation in the corresponding 3D cloaking structure is simulated by using the acoustic axial-symmetric module. A spherical cloaking effect is achieved in the 3D system and the obtained pressure field is presented in Fig. 8.

## Discussion

In summary, the self-focusing and directional cloaking properties of a 2D GRIN perforated aluminum slab are demonstrated in this paper. A 3D axisymmetric cloaking structure is obtained by rotating the corresponding 2D structure around the slab axis. By tailoring the matching coefficient and the anisotropy coefficient, a low-loss directional cloaking device is developed to guide the acoustic waves propagating around the central hollow area of the slab. The difficulty with the phase discontinuity inside and outside the slab is overcome by introducing a non-gradient slab behind the GRIN slab as an acoustic delay device. In addition to the self-focusing and directional cloaking properties, the present GRIN-PC structure may also be utilized in many other applications such as acoustic imaging, and directional emission.

## Method

Throughout the paper, all propagation wave simulations are performed by using COMSOL Multiphysics with the acoustic-solid interaction module. The materials applied in our simulations are water for the surrounding liquid and aluminum for the slab. The plane wave radiation boundary condition is adopted on the outer boundaries to eliminate the reflected waves. The incident wave beam in Fig. 3 is Gaussian type with the pressure distribution along the *y* axis as ${p}_{y}=exp(-{y}^{2}/{{\omega }_{0}}^{2})$.
